# Artificial Intelligence for Classifying and Archiving Orthodontic Images

**DOI:** 10.1155/2022/1473977

**Published:** 2022-01-27

**Authors:** Shihao Li, Zizhao Guo, Jiao Lin, Sancong Ying

**Affiliations:** ^1^National Key Laboratory of Fundamental Science on Synthetic Vision, College of Computer Science, Sichuan University, Chengdu, Sichuan 610041, China; ^2^Sichuan Hospital of Stomatology, Chengdu, Sichuan 610041, China; ^3^College of Computer Science, Sichuan University, Chengdu, Sichuan 610041, China

## Abstract

One of the main requirements for orthodontic treatment is continuous image acquisition. However, the conventional system of orthodontic image acquisition, which includes manual classification, archiving, and monitoring, is time-consuming and prone to errors caused by fatigue. This study is aimed at developing an effective artificial intelligence tool for the automated classification and monitoring of orthodontic images. We comprehensively evaluated the ability of a deep learning model based on Deep hidden IDentity (DeepID) features to classify and archive photographs and radiographs. This evaluation was performed using a dataset of >14,000 images encompassing all 14 categories of orthodontic images. Our model automatically classified orthodontic images in an external dataset with an accuracy of 0.994 and macro area under the curve of 1.00 in 0.08 min. This was 236 times faster than a human expert (18.93 min). Furthermore, human experts with deep learning assistance required an average of 8.10 min to classify images in the external dataset, much shorter than 18.93 min. We conclude that deep learning can improve the accuracy, speed, and efficiency of classification, archiving, and monitoring of orthodontic images.

## 1. Introduction

Image data are fundamental in most medical settings. In dentistry, for example, imaging is useful for diagnosis, treatment planning, monitoring, and doctor-patient communication. Orthodontists use image data for clinical decision-making, tracking teeth, and planning treatment. Traditionally, these images have been indexed (i.e., labeled based on clinical features) and stored manually, but as digital dentistry has advanced, imaging data are increasingly indexed and stored in digital archives or patient management systems, allowing for easy retrieval for further diagnostics, treatment, and monitoring [1]. Therefore, it would be useful to develop a fully automated classification and archiving method to improve the quality of dental work, as well as relieve the workload for orthodontists.

Image indexing is an image classification task that can be automated using artificial intelligence (AI), especially AI based on deep learning [2]. Deep learning is a branch of machine learning that excels in analyzing high-dimensional data such as text and images [3]. Deep learning has completely replaced certain traditional machine learning-based tasks in computer vision, such as classification [4], segmentation [5], and detection [6]. In dentistry, studies have begun applying deep learning to diagnosis, screening, and decision-making [7]. For example, one study [8] used deep learning to assist orthodontists in skeletal classification using a large dataset of lateral cephalograms (5890 images). After training and validating the model, those authors reported that their deep learning model performed vertical and sagittal skeletal classification with sensitivity, specificity, and accuracy of >90%. Another study reported a deep learning method that was able to detect dental caries in near-infrared transillumination imaging with an overall mean intersection-over-union score of 72.7% relative to the performance of professional dentists [9]. Additionally, deep learning can be used to automatically identify landmarks in X-ray images for the analysis of orthodontic treatments [10]. However, the imaging data in that study had to be manually selected from case data, as required in commonly used dental applications such as the Invisalign (Align Technology, Santa Clara, CA, USA) orthodontic system. Therefore, developing an automated classification, archiving, and monitoring method that can work in conjunction with other special analysis algorithms may lead to an end-to-end dental AI application that can improve the quality of clinical practice.

**Figure 1:**
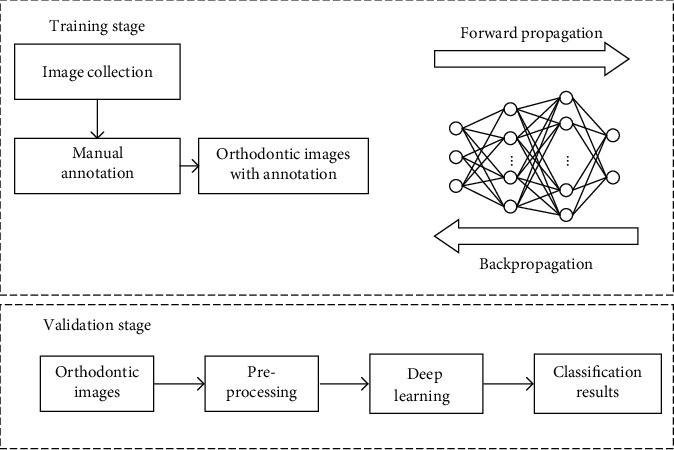
The construction of a deep learning model.

In the present study, we propose an automated deep learning method for the classification and archiving of orthodontic images based on the DeepID model [11], which leverages deep convolutional networks (ConvNets) to extract features and joint Bayesian [12] algorithm for verification. For practical application, this framework is also easy to extend new functions without retraining the model, because the classification result is obtained by comparing DeepID features of sample.  Figure 1 depicts a standard flowchart for the construction of deep learning model. A total of 15,819 orthodontic images were collected for model training, validation, and testing. A comprehensive evaluation of our model showed that we were able to accurately classify orthodontic images into six different intraoral photos, six different extraoral photos, and two radiographs. We also conducted experiments to make a comparison of our method and several popular models, such as ResNet-34 [13], GoogLeNet [14], and MobileNetV2 [15]. The results showed that although our model is relatively shallower, we still have achieved an excellent performance of 99.4% accuracy. Furthermore, our model was able to detect repeated or missing images in case data. As far as we know, this is the first report of an AI method to classify and archive orthodontic images. Our findings suggest that deep learning models can reduce tedious and repetitive work as well as improve the quality of orthodontic treatment, making AI a powerful tool for clinical practice.

## 2. Materials and Methods

### 2.1. Study Population

We retrospectively examined orthodontic images obtained from 1000 patients who received orthodontic treatment between January and December 2019 in the Sichuan Hospital of Stomatology, the Simai Clinic, and the Yingke Clinic. In order to evaluate our method, orthodontic images from 100 patients at the Haoya Clinic were obtained as an external dataset. Demographic and clinical characteristics of the patients included in the study are shown in Table 1.

### 2.2. Image Dataset

**Figure 2:**
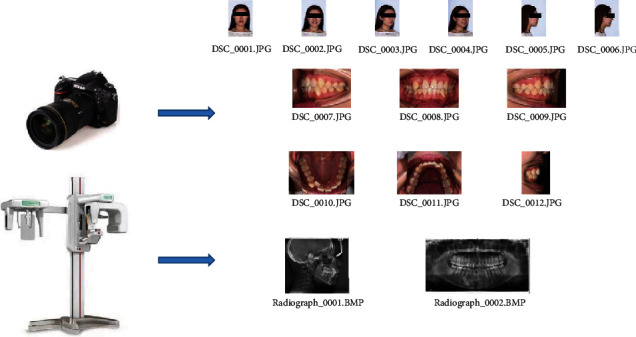
Diagram of data collection.

**Figure 3:**
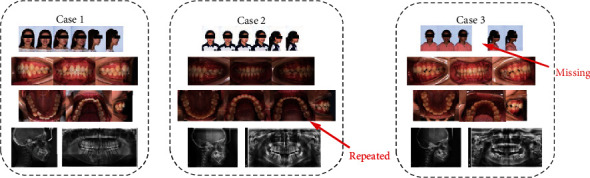
Representative examples of orthodontic images obtained from patients.

In this study, orthodontic images were defined as 14 categories: frontal at rest, frontal smile, oblique at rest, oblique smile, profile at rest, profile smile, intraoral right, intraoral front, intraoral left, maxillary occlusal, mandibular occlusal, overjet, lateral cephalogram, and panoramic radiograph. Data collection is shown in Figure 2. Representative examples of orthodontic images obtained from patients are shown in Figure 3.

Using these images, we created two nonoverlapping datasets: one was used as an internal dataset for model training and validation, and another was used as an external dataset to compare and evaluate the efficacy of human experts (orthodontists) versus the deep learning method. In both datasets, all orthodontic images were manually classified by an experienced orthodontist. To avoid mislabeled data and ensure the reliability of the dataset, a more senior orthodontic specialist with 30 years of experience reexamined all the images.

The original image was archived based on the patient list using unlabeled images. We found that half of the patients in the external dataset had repeated and/or missing images (~2 repeated and/or missing images per patient), and the remaining patients had a total of 14 qualified orthodontic images.

### 2.3. Classification of Orthodontic Images Based on Deep Learning

**Figure 4:**
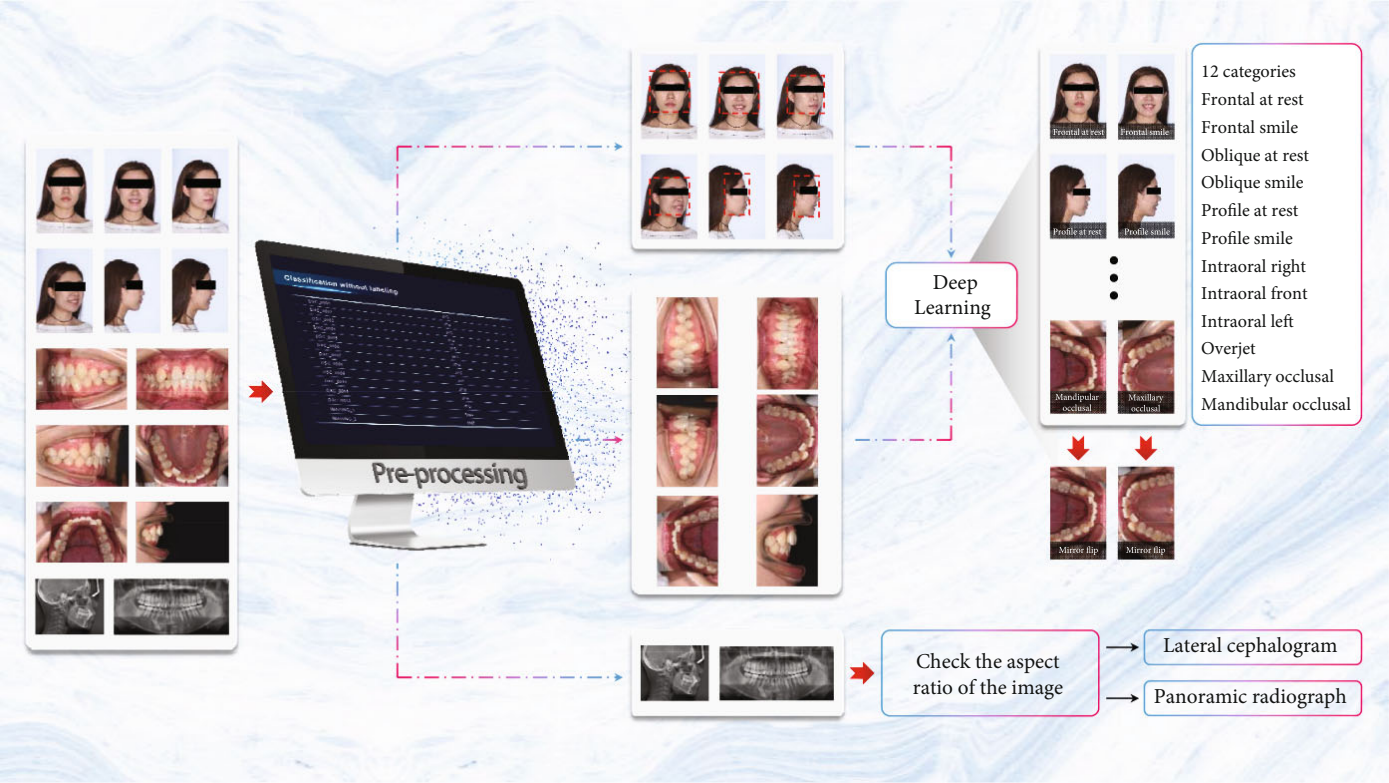
Flowchart depicting classification of orthodontic images using deep learning.

In this study, we propose a method of orthodontic image classification based on DeepID [11] that comprises three stages: preprocessing, classification, and postprocessing. All RGB images were checked and resized to 450 × 300 or 300 × 450 pixels based on their aspect ratio. A flowchart depicting the orthodontic image classification based on DeepID is shown in Figure 4.

The preprocessing stage included three functions: face detection, intraoral image transposition, and grayscale image tagging. The face detector was powered by OpenCV using the single-shot multibox detector (SSD) method [16]. In the case of dental imaging, the lateral cephalogram and the panoramic radiograph are typical grayscale images. Grayscale images are “one-channel”, and other images are RGB image with “three-channel”. Therefore, the grayscale images can be found easily available because of their “one-channel” characteristic. The final outputs of the preprocessing stage were facial regions, transposed original images, and grayscale images, if included.

In the next stage, the deep learning model processed the facial regions and the transposed photographs to classify each RGB image based on 12 categories. In addition, the grayscale images were examined in terms of their aspect ratio: the aspect ratio (width : height) of the lateral cephalogram was approximately 1.2 : 1, and that of the panoramic radiograph was approximately 2 : 1. Thus, classifying these images was straightforward. The deep learning model was trained using facial regions observed in intraoral photographs corresponding to 12 categories; these images were annotated by an orthodontist based on the guidelines provided by the orthodontic naming rule [17].

**Figure 5:**
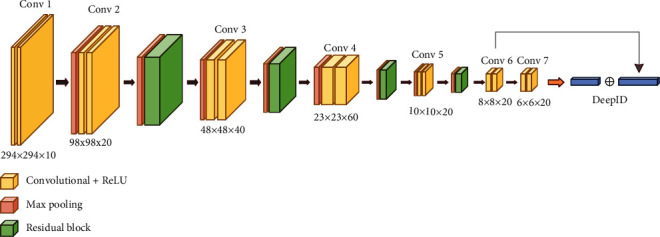
The architecture of the proposed model.

We designed our framework based on the concept of DeepID, which are high-level overcomplete features that contain discriminative information for recognition; after DeepID features have been produced, the joint Bayesian model will make classification based on them. The illustration of our DeepID features extraction process is shown in Figure 5.

**Figure 6:**
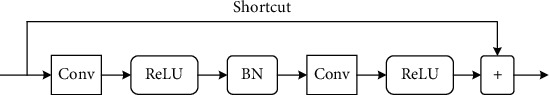
The structure of residual block.

Our framework is composed of convolutional layers, subsampling layers, ReLU layers, and residual blocks, as shown in Figure 5. In the method, we designed most of the convolutional functions with 3 × 3 filters; while concerning the images that fed into the network often with a larger size, we adopt 7 × 7 filters for the input layer. For improving convergence and reducing overfitting, we applied residual shortcuts after the Conv2 layer, Conv3 layer, Conv4 layer, and Conv5 layer, respectively. All residual blocks with the same architecture are illustrated in Figure 6. In the end, DeepID features were obtained based on the output of the Conv6 layer and Conv7 layer with a skip connection.

The residual architecture was proposed to address the issue of vanishing/exploding gradients and degradation that happened in traditional CNNs. After the inference step, produced DeepID features are passed to the joint Bayesian model [12] and yield the final classification results.

During orthodontic treatment, photographs of the maxillary and mandibular occlusal are obtained using an intraoral mirror, and orthodontists have to manually flip these images in order to analyze them further. Missing and repeat orthodontic images also frequently occur, making analysis even more inconvenient. In this study, we performed a mirror flip operation and an integrity check during the postprocessing stage based on the results obtained in the classification stage. Finally, experienced orthodontists confirmed the results of the deep learning model classification and, if necessary, corrected them for later orthodontic analysis.

### 2.4. Statistical Analysis and Evaluation Criteria

**Figure 7:**
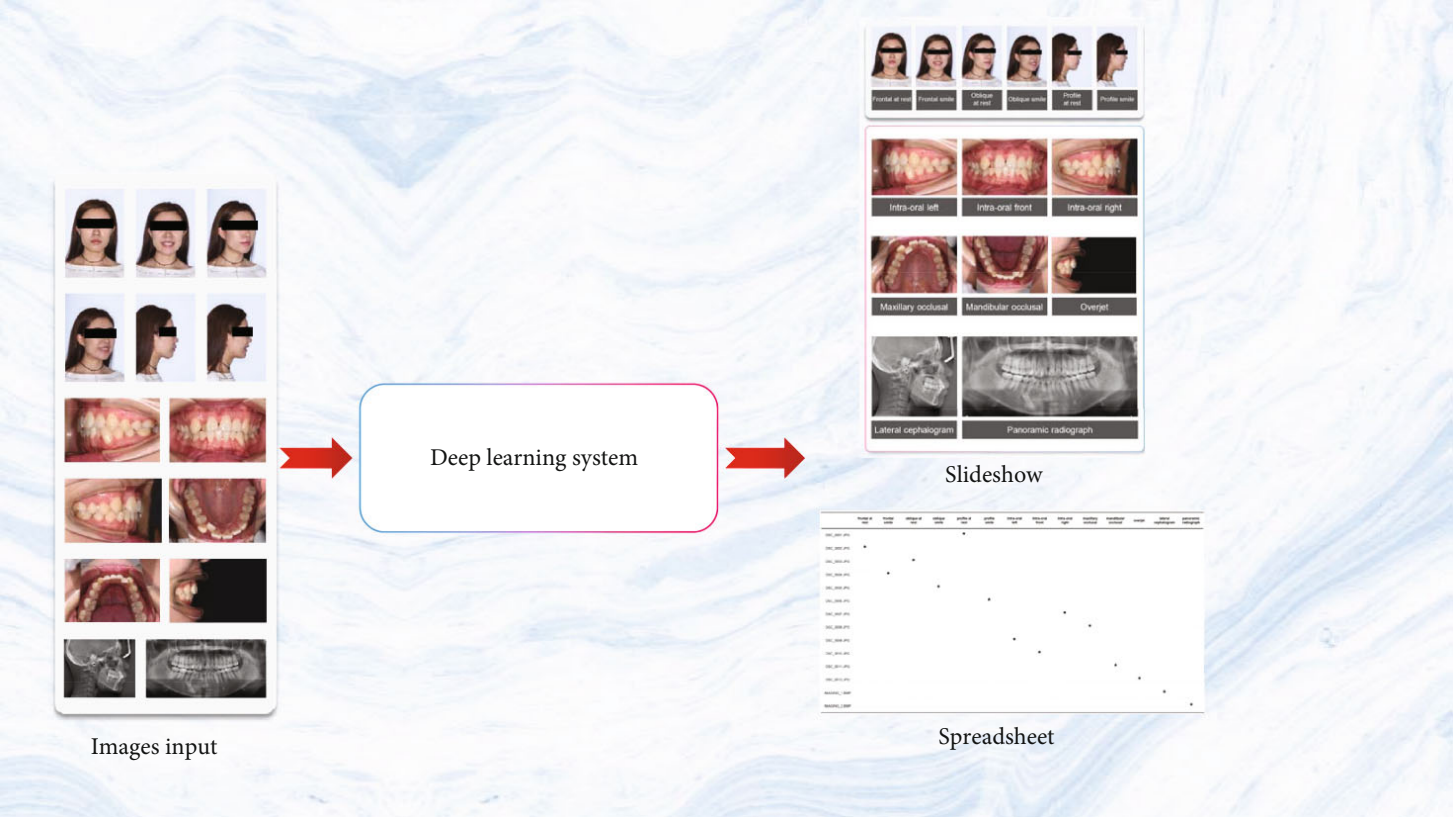
Overview of the classification and monitoring application.

**Figure 8:**
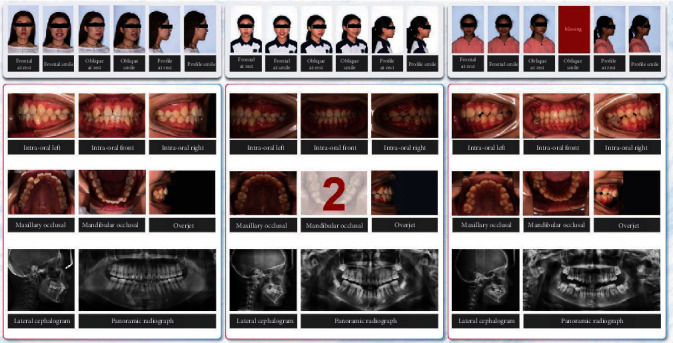
Slideshow examples of classification and monitoring of orthodontic images using deep learning.

All statistical analyses were performed using SPSS 26.0 (IBM, Chicago, IL, USA) and the Python sklearn library. To evaluate the performance of our method, we used the following metrics: accuracy, macro area under the curve (macro-AUC), time taken to archive, and receiver operating characteristic (ROC) curves. To compare the efficacy of the deep learning method against that of human experts, we compared the classification performance of three orthodontic specialists with more than five years of experience with that of the deep learning model on the same set of orthodontic images from the external dataset. The three specialists had been trained to identify images using orthodontic naming conventions [17]. In our AI system, deep learning generated an archiving spreadsheet that showed predictive classification and hyperlinks for each image (Figure 7), and the orthodontists had to confirm whether the classification generated by the deep learning model was consistent with their interpretation or not. In the case of inconsistencies, they corrected the classification of those particular images. If there were duplicate images in certain categories, the specialists selected one image that could be retained for that category. In addition, the specialists recorded missing images and categories (Figure 8).

## 3. Results

### 3.1. Imaging Dataset

A total of 16,221 orthodontic images were obtained from the included patients. Of these, we excluded blurred images (106) as well as other photographs and radiographs (296) that did not meet the requirements of the American Board of Orthodontists [18]. We included a total of 14,399 orthodontic images in the internal dataset and 1,420 orthodontic images in the external dataset. The internal dataset was then randomly divided into two groups: a training set (12,999 images) and a validation set (1400 images; 100 images corresponding to each of the 14 categories).

### 3.2. Deep Learning Model

All experiments were performed using Python 3.6 and TensorFlow 1.9 on a single NVIDIA RTX 2080Ti [19]. We proposed a modified model for automated classification, archiving, and monitoring of orthodontic images based on DeepID. In the training phase, we randomly selected 100 patients from the internal dataset as a validation set and performed a cross-validation procedure. Regarding the configuration of the hyperparameter, we used a learning rate of 0.001 and a batch size of 50 in the Adam optimizer. “Cross-Entropy” was chosen as the loss function, and the epoch number was set to 100 for model training. According to the performance of the validation set, the highest performance with respect to image classification occurred between 45 and 60 epochs. We selected the model based on the validation set with the highest performance for all subsequent work. Figure 8 shows slideshow examples of automated classification for orthodontic images according to the human-reviewed archiving table.

**Figure 9:**
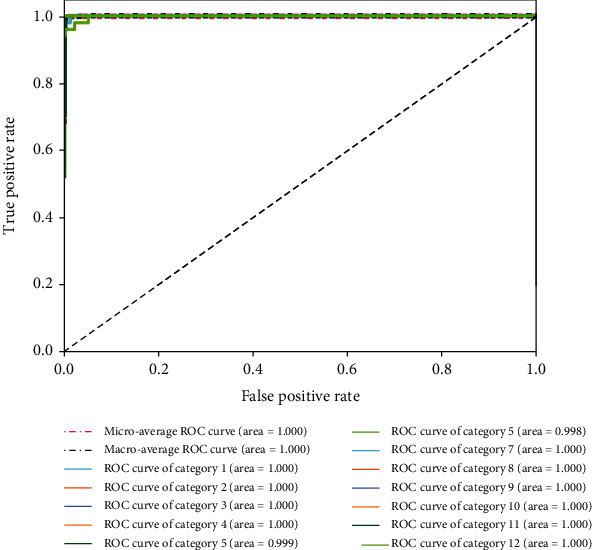
Receiver operating characteristic (ROC) curves associated with the deep learning model. Areas under the curve (AUCs) are provided in parentheses.

**Figure 10:**
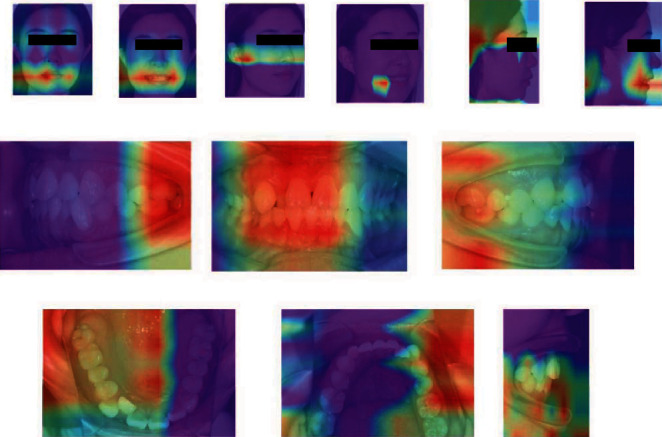
Gradient-weighted class activation maps (heat maps) highlighting regions in orthodontic images that were particularly relevant for classification.

The deep learning model was able to classify images within 0.08 min at an accuracy of 0.994 and a macro-AUC of 1.00. The ROC curves of our model are depicted in Figure 9, including macro- and micro-AUC, as well as ROC curves of all 12 categories. Although deep learning is considered to be a “black box”, gradient-weighted class activation mapping (Grad-CAM) can provide an explanation for the way in which deep learning systems make decisions based on their interpretation of the input data [20]. Grad-CAM provided visualizations of the weighted activation maps in the form of heat maps that highlight active regions of an image that were most relevant to the classification results (Figure 10).

### 3.3. Comparison of Advanced Deep Learning Models

We have conducted experiments on different models as well as other machine learning methods to make the evaluation, as illustrated in Table 2 and Table 3. We compared them by applying the metrics of parameter numbers, classification accuracy, and operation efficiency. For all models, we set input size as 300 × 300 × 3, and the Python package thop is applied to calculate the floating point operations per second (FLOPs). We can easily observe that GoogLeNet and our model achieved the highest accuracy, while our model requires the least parameters. In comprehensive consideration, our model can make precise recognition with less computational resources; it is significant for the application field.

### 3.4. Comparison of Model-Only and Expert-Only Classification

The deep learning model demonstrated a strong ability to learn from features in the radiographs, as well as from manually annotated intra- and extraoral images. Compared to expert-only classification, our model showed excellent performance and high accuracy for archiving orthodontic images (Table 4). Although the values of accuracy and macro-AUC were similar for the deep learning model and the human experts, we found that the deep learning model required only 0.08 min to archive 100 orthodontic patients (1,420 images), while a human expert required an average of 18.93 min to classify, select, remove, and record the same set of orthodontic images (Table 4). Our results indicate that the fully automated method based on deep learning was 236 times faster than the human expert.

### 3.5. Comparison of Human Experts with or without Deep Learning Assistance

To comprehensively evaluate the applicability of our deep learning model, we compared the efficiency of human-only and human-machine methods to classify, select, remove, and record orthodontic images. Three human experts with deep learning assistance required on average 8.10 min to classify and monitor images from the external dataset (100 patients), which was more efficient than manual classification performed by the human expert (18.93 min). Deep learning assistance also improved the accuracy of classification by 1% and the macro-AUC value by 0.1 (Table 4).

## 4. Discussion

Since orthodontic treatment requires continuous image acquisition, orthodontists have begun implementing automated classification and monitoring systems based on deep learning algorithms. The average length of orthodontic treatment can last anywhere between 12 and 36 months. All treatment begins with one or two initial consultations with an orthodontist, during which the orthodontist takes radiographs and photographs of patients, discusses the treatment options, and provides a detailed plan. However, during traditional acquisition of photographs and radiographs, missing and repeat orthodontic images frequently occur, making manual data archiving necessary for every patient. In the present study, we propose a practical deep learning-based method for the automated classification, archiving, and monitoring of orthodontic images. Our findings indicate that deep learning models can be used to quickly and effectively classify and monitor orthodontic images with very high accuracy, as well as support decisions about further orthodontic treatment.

Many studies have reported that deep learning methods have an impressive learning capacity and classification accuracy in dental applications, such as skeletal classification, detection of white spot lesions, and detection of dental caries [8, 25, 26]. However, very few studies have examined deep learning in the classification of orthodontic images. In the present study, we found that deep learning models can be used to effectively classify and monitor orthodontic images using a set of annotated photographs; the model tested in our study demonstrated excellent classification, as assessed using ROC curves and macro-AUC values. Additionally, the Grad-CAM heat maps indicated that our deep learning model, working only from image-level annotation, was able to identify differences in features across orthodontic categories. The heat maps in our study highlighted regions in the mouth, ear, and retractor as particularly relevant to classification. In addition, human experts with deep learning assistance classified orthodontic images with higher accuracy and efficiency than experts on their own.

In the present study, images of each orthodontic patient included six intraoral photographs, six extraoral photographs, and two radiographs. A study involving dental radiographs applied deep learning models to classify panoramic, periapical, bitewing, and cephalometric radiographs into four categories for image indexing and storing [27]: they found that deep learning showed superior performance in the classification task, with an accuracy of 99.7%, but they did not monitor the occurrence of repeated or missing images. In contrast to that work, we recommend classifying lateral cephalograms and panoramic radiographs using a computer program and clear classification rules if the aspect ratio is significantly different between lateral cephalogram and panoramic radiograph. However, the aspect ratio of radiographs is based on the radiograph machine so that the aspect ratio does not always exist significant differences. The ratio-based method may be ineffective if the aspect ratio is not significantly different between lateral cephalogram and panoramic radiograph. Under this condition, it is necessary to consider the deep learning method proposed by Cejudo et al. for radiograph classification [27]. Hence, we concluded that ratio-based method is more suitable for the radiographs with significant difference in aspect ratio, but deep learning as the second choice is also considered for the classification if the aspect ratio without significant differences. In addition, other machine learning methods (BCAoMID-F and CPoAMoTI) were compared to our deep learning model (Table 3). The experimental results demonstrated that traditional machine learning methods cannot accurately distinguish orthodontic images due to their limited capacity of feature extraction.

Our model takes advantage of residual architectures, which successfully prevented the problem that the model does not converge on the learning process due to vanishing/exploding gradients. The proposed model is quite small compared to advanced methods, so we can avoid many problems, like overfitting, the limitation constrained by computational resources [28]. A small model also leads to a fast recognition speed. It helps the real-time application. The model is custom-made for a certain target, and the size and architecture of it balanced the accuracy and speed. Therefore, after plenty of parameters adjust work, it is superior to these advanced models on this kind of orthodontic image recognition task. For the task of fixed-number categories, DeepID-based method does not show superiority relative to other classification models, but concerning the expansibility, the produced DeepID features can directly transfer for other tasks without retraining network; this is significant for practical application.

We are unaware of previous studies using deep learning to classify extraoral images. We speculated that deep learning models cannot effectively learn features from extraoral images if they are trained using images at the original resolution. Indeed, our model also showed unsatisfactory performance when asked to classify extraoral images at their original resolution. Studies on face recognition show that developers prefer to train deep learning models using facial regions within images, rather than the entire images [29]. Differences among facial regions are usually visible in the regions of the mouth, ears, and facial wrinkles. However, the resolution in these regions can be much smaller than the resolution of the original image, so the model may find it difficult to learn the relevant features. In order to overcome this difficulty, we made sure that facial regions were detected and cropped to identify feature constraints; these facial regions were then used for model training and testing for the classification of extraoral photographs. According to our experimental results, deep learning showed high accuracy in the classification of extraoral photographs when the facial region detector was used.

As far as we know, the present work is the first study testing a deep learning model for the classification, archiving, and monitoring of orthodontic images. Many popular orthodontic systems still use manual classification methods for archiving and managing patient data: our proposed method can be effectively integrated into these applications to help orthodontists save time and effort. Our findings show that the differences among orthodontic images are large enough that deep learning can easily classify them. In fact, we were able to identify all 14 categories of orthodontic images using our model. We also demonstrated that deep learning is a superior and promising method as a useful tool for dental practice. And further validation is still required by using different types of datasets from different sources, different practices, and different regions across the world.

As digital dentistry has advanced, many dental applications have been developed for the automated analysis of dental imaging. As a fundamental yet flexible method, our deep learning approach can help these dental applications quickly find the required data among a massive number of orthodontic images. For example, deep learning can be used to detect and localize malocclusion in intraoral photographs [30], and it can assess facial attractiveness based on extraoral photographs [31]. Deep learning can also extract features from radiographs and then identify landmarks or detect disease in an automated way [7, 10]. In future, it may be possible to apply deep learning to even more complex tasks, such as angle's classifications of malocclusion.

Nevertheless, our study presents several limitations. Firstly, our results must be considered with caution in light of the fact that our method was based on orthodontic-required images. We applied the model only to images that experienced orthodontists had manually reviewed in order to ensure adequate quality and appropriateness. Hence, our model may not achieve enough high accuracy in other datasets which exist significant differences with our dataset. Secondly, the performance of deep learning mainly relies on massive training samples with high-quality annotation. However, the manual annotation is a labor-intensive work, especially in dentistry. Thus, annotation for model training may not carry out in some geographical areas because of the lack of dentists. Finally, deep learning is a data-driven method so that the quality of massive sample is required to be controlled by human experts. Future work should explore automated quality evaluation of images prior to classification, which will be especially important for processing extremely large datasets.

## 5. Conclusions

In this paper, a deep learning model was developed for classifying and archiving orthodontic images based on DeepID. The performance of the model was comprehensively evaluated by an external testing set and comparison with orthodontists. Our findings show that deep learning methods can be used to automatically classify, archive, and monitor orthodontic images with higher accuracy and speed than manual methods. The modified model based on DeepID used in this study demonstrated an excellent ability to classify orthodontic images. Additionally, deep learning can help make dental follow-up and treatment more efficient, while reducing dentists' workload.

## Figures and Tables

**Table 1:** Clinical and demographic characteristics of included patients.

Characteristic	Internal dataset (*n* = 1,000)		External dataset (*n* = 100)
	Training (*n* = 900)	Validation (*n* = 100)	
Age in years	29 (4-62)	27.9 (4-58)	28.7 (4-60)
Sex			
Male	248 (27.6)	30 (30)	32 (32)
Female	652 (72.4)	70 (70)	68 (68)
Photograph classification			
Frontal at rest	950 (7.3)	100 (7.1)	110 (7.7)
Frontal smile	1,012 (7.8)	100 (7.1)	99 (7)
Oblique at rest	896 (6.9)	100 (7.1)	109 (7.7)
Oblique smile	906 (7)	100 (7.1)	123 (8.7)
Profile at rest	1,002 (7.7)	100 (7.1)	87 (6.1)
Profile smile	935 (7.2)	100 (7.1)	90 (6.3)
Intraoral right	976 (7.5)	100 (7.1)	102 (7.2)
Intraoral front	899 (6.9)	100 (7.1)	109 (7.7)
Intraoral left	1,009 (7.7)	100 (7.1)	107 (7.5)
Maxillary occlusal	828 (6.4)	100 (7.1)	98 (6.9)
Mandibular occlusal	932 (7.2)	100 (7.1)	97 (6.8)
Overjet	854 (6.6)	100 (7.1)	89 (6.3)
Radiograph classification			
Lateral cephalogram	900 (6.9)	100 (7.1)	100 (7)
Panoramic radiograph	900 (6.9)	100 (7.1)	100 (7)
Total number of images	12,999	1,400	1,420

Values are *n*, *n* (%), or median (range).

**Table 2:** Comparison of our algorithm with several popular models.

Method	Parameters (M)	Accuracy (%)	Efficiency (MFLOPs)
AlexNet [21]	57.1	98.2	1198.7
GoogLeNet [14]	5.6	99.4	2589.1
MobileNet V2 [15]	2.2	98.7	587.5
ResNet-34 [13]	21.2	99.2	6849.5
DenseNet-121 [22]	6.9	98.2	4991.4
ShuffleNet V2 [15]	0.35	97.3	**78.6**
Ours	**0.17**	**99.4**	211.9

**Table 3:** Comparison of our model and other machine learning methods.

Method	Accuracy (%)
BCAoMID-F [23]	84.3
CPoAMoTI [24]	77.2
Ours	99.4

**Table 4:** Performance of human experts and deep learning during classification of orthodontic images in the external dataset.

Operator	Method	Accuracy	Macro-AUC	Time taken to archive (min)
Deep learning	Automatic	0.994	1.00	0.08
Expert 1	Manual	0.988	0.992	19.19
	Deep learning assistance	0.998	0.997	8.27
Expert 2	Manual	0.987	0.985	18.97
	Deep learning assistance	0.997	0.996	7.92
Expert 3	Manual	0.983	0.983	18.63
	Deep learning assistance	0.996	0.996	8.10

AUC: area under the curve.

## Data Availability

The datasets generated or analyzed during the current study are not publicly available in order to preserve patient confidentiality but are available from the corresponding author on reasonable request.
